# Premature birth associated with a favorable course in gestational alloimmune liver disease (GALD): A case report

**DOI:** 10.3389/fped.2023.1104530

**Published:** 2023-03-16

**Authors:** Linda-Marie Mulzer, Heiko Reutter, Jörg Jüngert, A. S. Knisely, Margit Schmid, André Hoerning, Patrick Morhart

**Affiliations:** ^1^ Department of Pediatrics, University of Erlangen-Nürnberg, Erlangen, Germany; ^2^ Diagnostic and Research Institute of Pathology, Medical University of Graz, Graz, Austria; ^3^ European Reference Network on Hepatological Diseases (ERN RARE-LIVER), ZSEER, University Hospital Erlangen, Friedrich-Alexander-University of Erlangen-Nuremberg, Erlangen, Germany

**Keywords:** gestational alloimmune liver disease, liver disease, neonatal acute liver failure, prematurity, neonatal hemochromatosis, prematurit

## Abstract

Gestational alloimmune liver disease (GALD) is a rare neonatal disorder with high mortality and morbidity. The patients come to caregivers' attention aged a few hours or days. The disease manifests as acute liver failure with or without siderosis. The differential diagnosis of neonatal acute liver failure (NALF) is broad, including mainly immunologic, infectious, metabolic and toxic disorders. The most common cause, however, is GALD followed by herpes simplex virus (HSV) infection. The best suited pathophysiological paradigm of GALD is that of a maternofetal alloimmune disorder. State of the art treatment combines intravenously administered immunoglobulin (IVIG) with exchange transfusion (ET). We report an infant born at 35 + 2 weeks' gestation in whom GALD had a favorable course, of interest because premature birth in our patient may have exerted protective aspects and lessened morbidity in that intrauterine exposure to maternal complement-fixing antibodies was shortened. The diagnosis of GALD was challenging and difficult. We suggest a modified diagnostic algorithm combining clinical findings with histopathologic findings in liver and lip mucosa and, if available, on abdominal magnetic resonance imaging-study focusing on the liver, spleen, and pancreas. This diagnostic workup must be followed by ET and subsequent administration of IVIG without delay.

## 1. Introduction

Gestational alloimmune liver disease (GALD) is a rare neonatal disorder with high mortality and morbidity (1). The phenotype of GALD is broad. Antenatal findings can include fetal growth retardation, oligohydramnios, fetal and placental edema, and maternal history of stillbirth or miscarriage (2–4). Postnatally GALD manifests as acute liver failure with or without hepatic or extrahepatic siderosis (2). Patients come to caregiver attention aged a few hours or days with jaundice, sepsis-like symptoms, and hyperacute neonatal liver failure (NALF). The Pediatric Acute Liver Failure Study Group include coagulopathy as the fundamental criterion when diagnosing ALF in children (5). They define coagulopathy as prothrombin time >20 s or an international normalized ratio (INR) ≥2 following the administration of parenteral vitamin K. Further clinical signs comprise food refusal, hypoglycemia, and jaundice. Clinical-laboratory biomarkers include hypoalbuminemia, high serum ferritin values, hypotransferrinemia with hypersaturation of available transferrin, concomitant and nonspecific high tyrosine levels, and marked and multifactorial coagulopathy unresponsive to vitamin K. Serum transaminase levels in GALD are usually only slightly elevated (and sometimes even normal), which may help in differentiating it from other causes of acute liver failure. Another typical sonographic and histopathologic feature of GALD is liver fibrosis, which is absent in most other causes of NALF, *e.g.*, acute viral infections. In addition, a persistently patent ductus venosus due to increased resistance within the hepatic sinusoidal bed resulting from advanced liver fibrosis can be observed by ultrasound (6, 7). NALF has a broad differential diagnosis, including immunologic, infectious, metabolic (including mitochondrial), toxic, and haemato-oncologic disorders, as well as severe congenital heart disease. Nevertheless, GALD is the most common cause of NALF, followed by herpes simplex virus (HSV) infection (5, 6).

GALD is a sporadically manifesting familial disorder for which no genetic predisposition has been identified. Most cases of “neonatal hemochromatosis” (NH) appear to represent GALD (6, 8). Because the phenotype of severe liver disease and multi-organ siderosis, sparing the reticuloendothelial system, matches that of advanced iron storage disease (now traced in most patients to *HFE* variants), NH was considered a primary disorder of iron handling (8). NH is now recognized as a clinical phenotype shared by various forms of liver disease of fetal onset, including GALD-NH, rather than as a disease itself. A corollary of interest is that instances of GALD without siderosis are reported (2).

A pathophysiological paradigm best suited to many instances of GALD is that of a maternofetal alloimmune disorder. In disease of this sort, the placenta actively transports maternal IgG-class antibodies into the circulation of the conceptus that are directed against an antigen expressed by the fetus. Injury mediated by complement fixation follows. The pattern of recurrence of GALD within sibships and the occurrence of GALD in maternal half-siblings are consonant with such a disorder. This paradigm was first proposed by Whitington, who hypothesized that in GALD mothers form antibodies against an antigen expressed by fetal hepatocytes (9). In a mother sensitized against such an antigen, maternal IgG-class antibodies attack fetal hepatocytes, activating the classical complement pathway. These events start around the 12th week of gestation (10, 11) and lead to hepatocyte death mediated by the complement membrane attack complex C5b9. The postulated hepatocellular antigen is still unknown (2, 6). The absence of maternal liver disease in GALD also remains puzzling; the antigen may exist only in the fetal liver or may be almost completely gone in the adult liver so that the maternal immune system lacks tolerance for this specific antigen (2).

GALD can be suspected if NALF is associated with a patent ductus venosus and hyperferritinemia, although both are rather non-specific findings. The frequently observed patency of the ductus venosus is ascribed to increased resistance within the hepatic sinusoidal bed due to fibrotic or even cirrhotic transformation of liver parenchyma. It is also described in trisomy 21 patients with liver fibrosis owing to transient abnormal myelopoiesis (12).

A more specific criterion might be the presence of extrahepatic siderosis that spares the spleen and lymph nodes. Extrahepatic siderosis may affect myocardium, pancreas, salivary glands, thyroid gland, and various epithelia at other sites. This reflects altered iron metabolism caused by loss of functional liver parenchyma followed by decreased synthetic capacity and leading to hypotransferrinemia and hypohepcidinemia (6, 13). Hypohepcidinemia, a result of loss of hepatocellular synthetic capacity, is suggested to perturb fetoplacental feedback regarding iron stores within the conceptus, thereby increasing transplacental iron uptake and causing absolute iron overload (13). Loss of functional liver mass additionally decreases iron storage capacity within fetal hepatocytes and leads to spill-over of iron into non-transferrin bound iron (NTBI). NTBI can enter cells (except erythroid cells) in an unregulated way and thereby disturbs intracellular iron balance and contributes to ferroptosis. The spillover of iron into NTBI converges with failure of iron-export downregulation in macrophages *via* hepcidin-controlled ferroportin. This yields siderosis of a variety of tissues with sparing of reticuloendothelial sites, the pattern seen in GALD (13).

A widely accepted diagnostic criterion for GALD is the identification of C5b9 complement deposits in a heavily inflamed and fibrotic liver (4, 14). Nevertheless, other authors (*e.g*., Yeh *et al.*) point out that the specificity of C5b9 staining is still arguable since non-GALD cases also may express C5b9 and that in some GALD cases C5b9 is not immunohistochemically demonstrable (1). In addition, biopsy of the mucosa of the lower lip to assess minor salivary glands for stainable extrahepatic iron deposits is a valuable diagnostic tool. The biopsy specimen can be obtained at bedside with only local anesthesia and hemostasis can be assessed and treated under direct vision (15–17). Beside tissue biopsy, magnetic-resonance imaging (MRI) can be used to confirm extrahepatic siderosis. Although in infants sedation is required, MRI is non-invasive and permits quick assessment of tissue iron stores, preferably in the pancreas and spleen—the spleen to demonstrate lack of iron within the reticuloendothelial compartment—using multi-echo sequence with increasing echo time (18).

Before alloimmune pathophysiology was hypothesized, with the paradigm of GALD, treatment of NALF diagnosed as NH consisted of antioxidant and chelation therapy. State-of-the-art treatment of GALD, arrived at by analogy, now consists of intravenously administered immunoglobulin (IVIG), 1 g/kg, with or without exchange transfusion (ET). Currently, a standardized treatment protocol does not exist. Further doses of IVIG, up to 5 g/kg within 2 weeks, may be given, and ET may be multiply repeated (1, 19). With this approach the historically high mortality rate of GALD, at 80%–90%, has been reduced substantially (2, 19). Since an effective treatment strategy is available, early therapy is decisive to achieve a good clinical outcome. Therefore, and to minimize morbidity and mortality, rapid diagnosis is essential. Feldman and Whitington suggest a flow-chart for diagnosis in suspected GALD (2). Making the diagnosis of GALD is important not just for guiding management but also for the treatment of future pregnancies. The high recurrence rate of GALD within sibships, at 67%–92% (20, 21), highlights the importance of antepartum treatment of an expectant mother who has previously borne an infant with GALD (22). When given to a pregnant woman, IVIG and plasmapheresis can successfully treat various diseases of the fetus, such as fetal-neonatal alloimmune thrombocytopenia, antiphospholipid syndrome, and hemolytic anemia due to maternofetal blood-group incompatibility (23–25). Therefore, administration of IVIG and plasmapheresis are also possible treatment options in GALD cases (22).

## 2. Case report

Informed written consent for publication of the case report, including pictures, was obtained from parents. We report an infant born at 35 + 2 weeks' gestation weighing 2020g (7th percentile); Apgar scores were 9/9/10. In the 20th week of pregnancy, the mother had developed pemphigoid gestationis (shown in Supplementary Figure S1), which was treated successfully with topical glucocorticoids. Pregnancy was further complicated by premature rupture of membranes. The placenta was not examined. The child was admitted to the neonatal department due to prematurity. Routine serum glucose checks detected hypoglycemia. Hypothyreosis and hypocortisolism as a cause for hypoglycemia were ruled out; newborn screening showed no abnormalities and results of blood gas analysis, including lactate levels, were unremarkable. On the 5th day of life, the baby manifested sepsis-like signs, reduced muscle tone, jaundice, and difficulties in feeding and appeared critically ill. Values for C-reactive protein, interleukin 6, and leucocyte count were determined several times and were unremarkable. Blood was cultured on several occasions; no micro-organisms grew. Despite intravenous glucose administration, hypoglycemia recurred. Hypocoagulability with a very low Quick of 17% was detected. Decreasing plasma concentrations of clotting factors and thrombocytopenia were also observed. Serum albumin was reduced (22.9 g/L, standard value >38.0 g/L). Hepatocellular synthetic failure was diagnosed. Transaminase activities and total serum bilirubin concentration were elevated (GOT maximum 649 U/L, normal range <96 U/L; GPT 91 U/L, <71 U/L). Total serum bilirubin concentration was 13.2 mg/dl (<8.7 mg/dl)). A serum ammonia value was within expected ranges. Vitamin K—unresponsive coagulopathy (INR 3.99 / Quick 17%) indicated NALF, meeting a criterion of the Pediatric Acute Liver Failure Study Group.

Findings of laboratory studies to address possible causes of NALF are shown in Table 1. Congenital infections were rapidly excluded; no testing was conducted regarding infections with rubella, *Listeria* sp, parvovirus B19, and others because of the clarity of the clinical findings pointing toward GALD; no history of exposure to drugs or hepatotoxins could be elicited from the mother. Amino-acid and organic-acid values in serum and urine were remarkable only for an elevated tyrosine value at 848.1 µmol/L (normal range < 200 µmol/L) without concomitant succinylacetonemia. Additionally, a ferritin value was increased (992 µg/L, <60 µg/L), as was a free serum iron value (194 µg/dl, <127 µg/L). A transferrin value was below normal range (1.32 g/L, 1.55–3.11 g/L). Transferrin saturation was 92.4%, a remarkably high value (at term following immediate postnatal equilibration <44%). These abnormalities fit the pattern observed in severe liver disease of fetal onset, suggesting GALD.

**Table 1:** Exclusion of infectious causes of NALF.

Pathogen	Material	Results	Interpretation
Herpes simplex virus (HSV) 1	Ethylene diamine tetraacetic acid—anticoagulated (EDTA) blood	HSV1 DNA negative	No sign of HSV1 infection
HSV 2	EDTA blood	HSV2 DNA negative	No sign of HSV2 infection
Cytomegalovirus (CMV)	Urine	CMV DNA quantitation, <250 copies/ml	No sign of CMV infection
Toxoplasma gondii	EDTA blood	IgG/IgM/IgA negative	No signs of congenital toxoplasmosis infection
Epstein-Barr virus (EBV)	EDTA blood	Anti-EBV viral capsid antigen IgM negative	Transplacentally acquired antibodies only (remote maternal EBV infection, no acute fetal EBV infection)
		Anti-EBV nuclear antigen IgG positive	
Hepatitis B virus (HBV)	EDTA blood	HBV surface antigen (HBs) negative	Transplacentally acquired antibodies only (remote maternal HBV infection, no acute fetal HBV infection)
		Anti-HBs antibodies present (26.2 IU/L)	
Hepatitis C	EDTA blood	HBc-IGM negative	No proof of HCV infection
		Anti-HCV antibodies negative	

On ultrasonography a hyperechogenic liver with textural abnormalities and patent ductus venosus was seen (shown in Supplementary Figures S2, S3). No edema or ascites was found. Hepatic vascular diseases and congenital heart disease were ruled out by ultrasonography and echocardiography.

**Figure 1:**
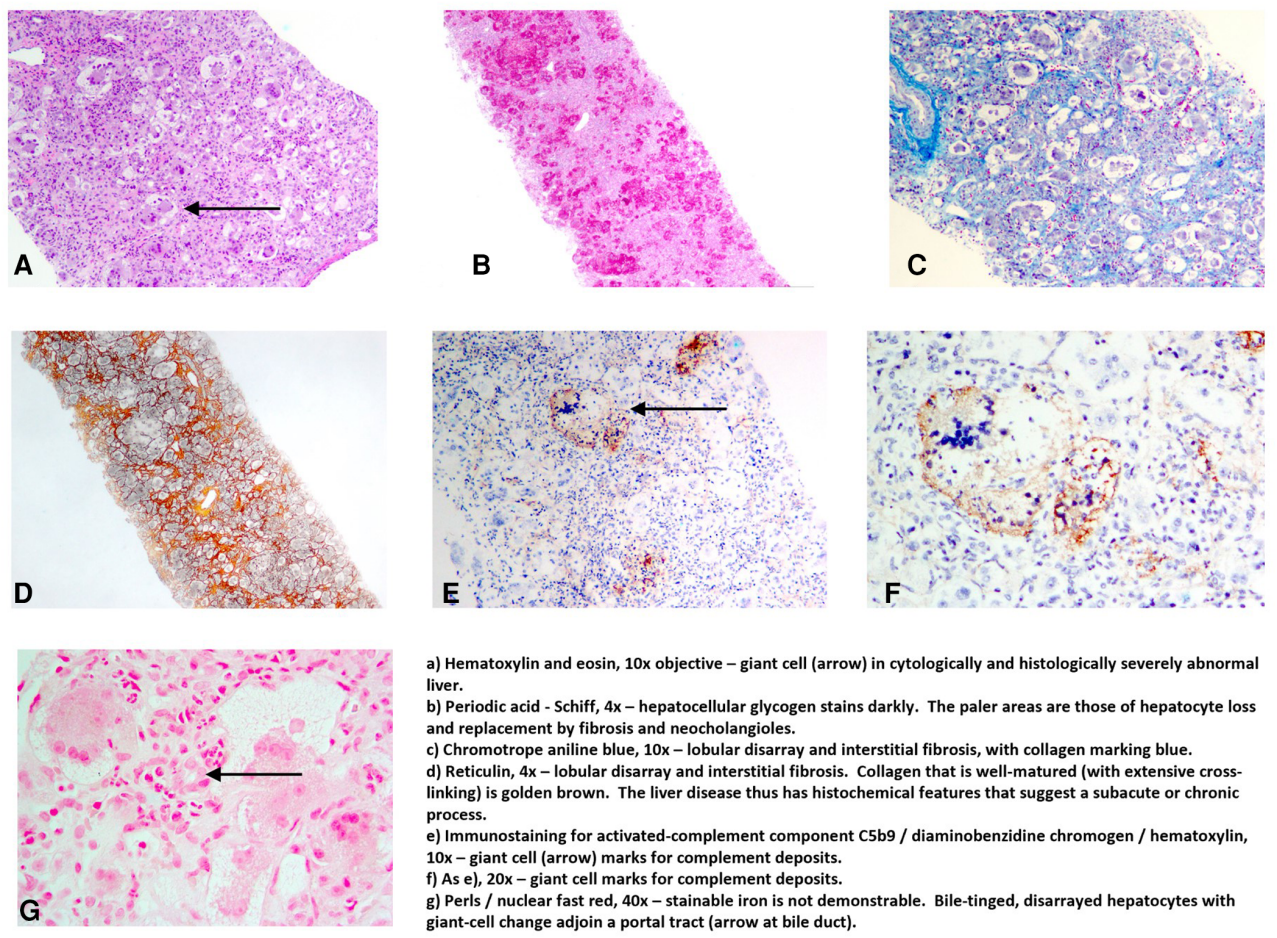
Photomicrographs, liver-biopsy specimen, A.S. Knisely, Graz.

**Figure 2:**
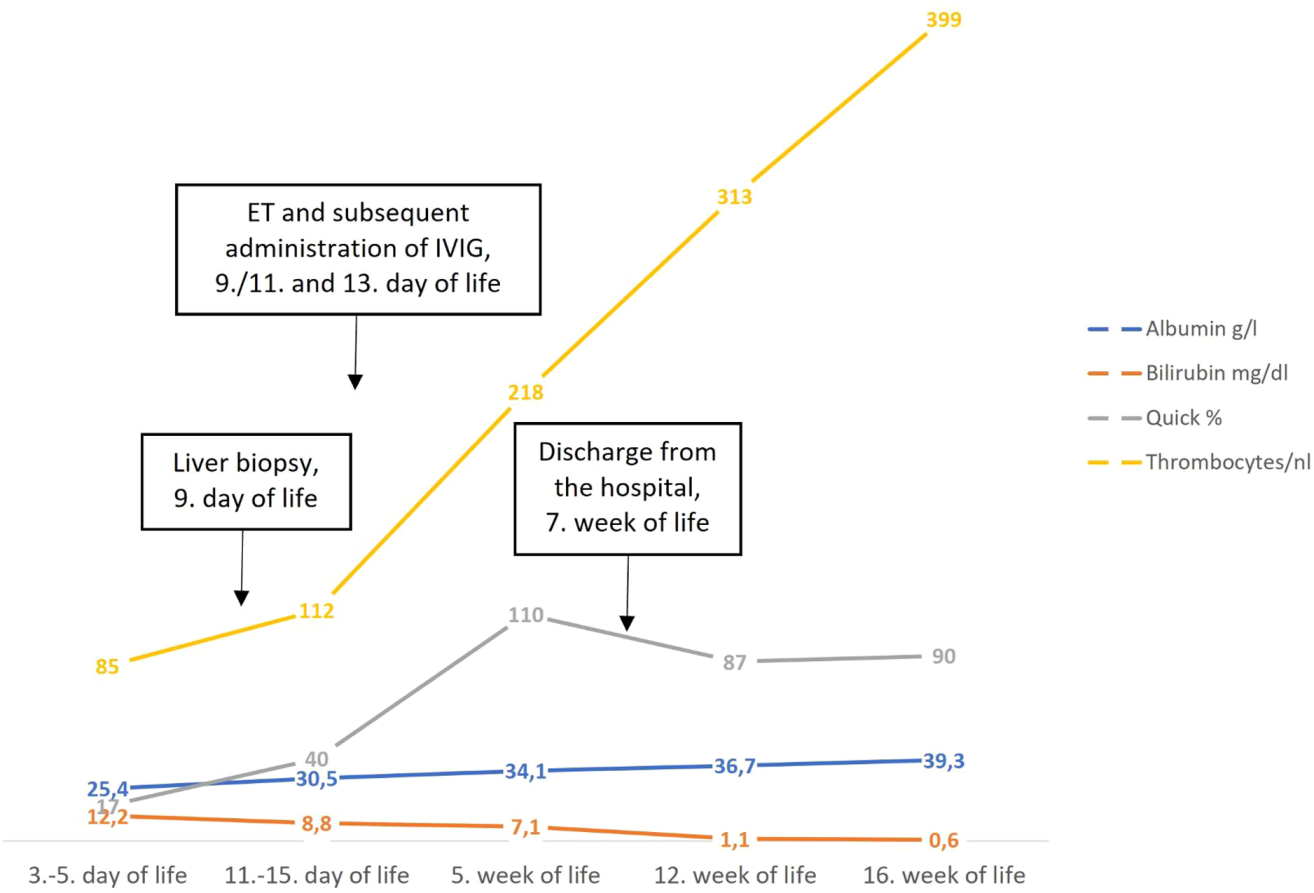
Normalization of laboratory findings after therapy. Supplementation of vitamin K was discontinued after 4 weeks. ET, exchange transfusion, IVIG, intravenous immunoglobulins.

The working diagnosis, given the clinical presentation and both laboratory and imaging-study findings (hypersaturation of transferrin with hepatocellular synthetic failure; small liver with patent ductus venosus; lack of evidence for infectious or usual metabolic disease), was GALD. To address the suspicion that NH in this patient was due to GALD and to direct therapy, a liver biopsy was conducted on postnatal day 9. Coagulation was optimized before the intervention by administration of blood products. Nevertheless, postinterventional bleeding occurred (detected by ultrasound, successfully treated conservatively). Light microscopy of the liver-biopsy specimen found giant-cell hepatitis with intralobular cholestasis, fibrosis, and parenchymal loss. Residual hepatocytes bore immunohistochemically demonstrable C5b9 deposits (Figure 1). The pattern of abnormalities was compatible with GALD. We refrained from performing a lip-mucosa biopsy as the clinical picture was very characteristic for GALD. MRI was not feasible in this severely ill newborn. Therapy was therefore directed at presumed GALD, initiated immediately after liver biopsy on postnatal day 9 (4 days after the diagnosis of NALF) with the first of three ET (80 ml/kg) each followed by IVIG (1 g/kg). ET with subsequent IVIG infusion was repeated on postnatal days 11 and 13. In addition, ursodeoxycholic acid and vitamins A, D, E, and K were given. The response was satisfactory, demonstrating normalization of liver-injury biomarker values and coagulation parameters within 5 weeks (shown in Figure 2). The patient tolerated enteral nutrition well and displayed normal growth in body weight and length. Neurologic development was assessed as normal. Clinical assessment at age 2 years also showed normal development with unremarkable biomarker values, requiring no further treatment. Sonography showed borderline hepatosplenomegaly with liver of unremarkable echogenicity and texture. These developments obviated liver transplantation, an option discussed early in the patient's course and discarded due to low body weight (practicable, if other therapies fail, at body weight 3–4 kg).

## 3. Discussion

Clinical presentation, clinical-laboratory findings, imaging-study findings, and the histological demonstration of activated-complement deposits in the damaged liver permitted diagnosis of GALD as the cause for NALF. The intrauterine environment is hostile when a maternal alloimmune disorder exists. This can be alleviated in part by intervention such as preventive IVIG administration to the mother starting in the 14th week of pregnancy if GALD previously occurred in a sibling or by preemptively removing the fetus by cesarian section from that environment. Premature birth in our patient may thus have exerted protective aspects and lessened morbidity because intrauterine exposure to maternal complement-fixing antibodies was shortened (2). However, prematurity itself can of course also have negative effects on the course of the disease; liver transplant, *e.g.*, is so technically difficult in small infants as not to be feasible. In any event, the patient responded well to ET and IVIG administration. Should the mother again embark on pregnancy, immunologic support and close monitoring will be offered.

The diagnosis of GALD is challenging and difficult. Following the suggested flow-chart by Whitington *et al.* (2), it mainly depends on the demonstration of extrahepatic siderosis by MRI or lip mucosa biopsy. Indeed, liver biopsies in neonates suffering from a severe coagulopathy, as in GALD, carry a high risk of bleeding and complications. Therefore, biopsy of the mucosa of the lower lip, to assess minor salivary glands for stainable iron, may be preferable (15, 16). However, cases of GALD causing liver failure without concomitant NH have been well documented and at least in some of these NALF cases this was not associated with hepatic and extra-hepatic siderosis (26). Hence, these are the cases in which a liver biopsy would be the essential diagnostic tool as they will be missed performing only lip mucosa biopsy and MRI.

In addition, MRI in neonates carries disadvantages. While the MRI technique for iron quantification in various organs is well established in older children and adults (27–29), data on quantification in neonates are limited (30). Furthermore, in many centers it is still a challenge to obtain good quality MRI images with optimal signal-to-noise ratio in small organs of distinctive interest like pancreas or thyroid (18, 29). To overcome these technical problems, the image quality needs to be optimized with small appropriate-sized surface coils for better signal and thin slices to visualize these small organs (27). Moreover, deep sedation is needed to prevent the baby from moving.

To detect C5b9 deposits at hepatocytes immunohistochemically is an alternate option for the diagnosis of GALD (2, 4, 14). Nevertheless, it is invasive, requiring a liver biopsy, and this may be delayed or not possible in the setting of liver failure-related coagulopathy. However, a liver biopsy includes additional benefits, such as information on the degree of hepatic fibrosis and the severity of inflammation. Moreover, further investigations to exclude differential diagnoses, for example virological examination, can be conducted easily and early. On the other hand, it has also become clear in recent years that GALD can produce liver disease without extrahepatic siderosis; that the absence of pathologic siderosis in the newborn liver does not exclude the diagnosis of GALD; and that to demonstrate C5b9 deposits at hepatocytes is not pathognomonic for GALD (2, 26, 31).

Therefore, a thoughtful correlation of clinical findings and histopathologic findings, including immunostaining of liver for C5b9 when possible, lip mucosa biopsy, and imaging-study results, is generally recommended in approaching the differential diagnosis (31).

**Figure 3:**
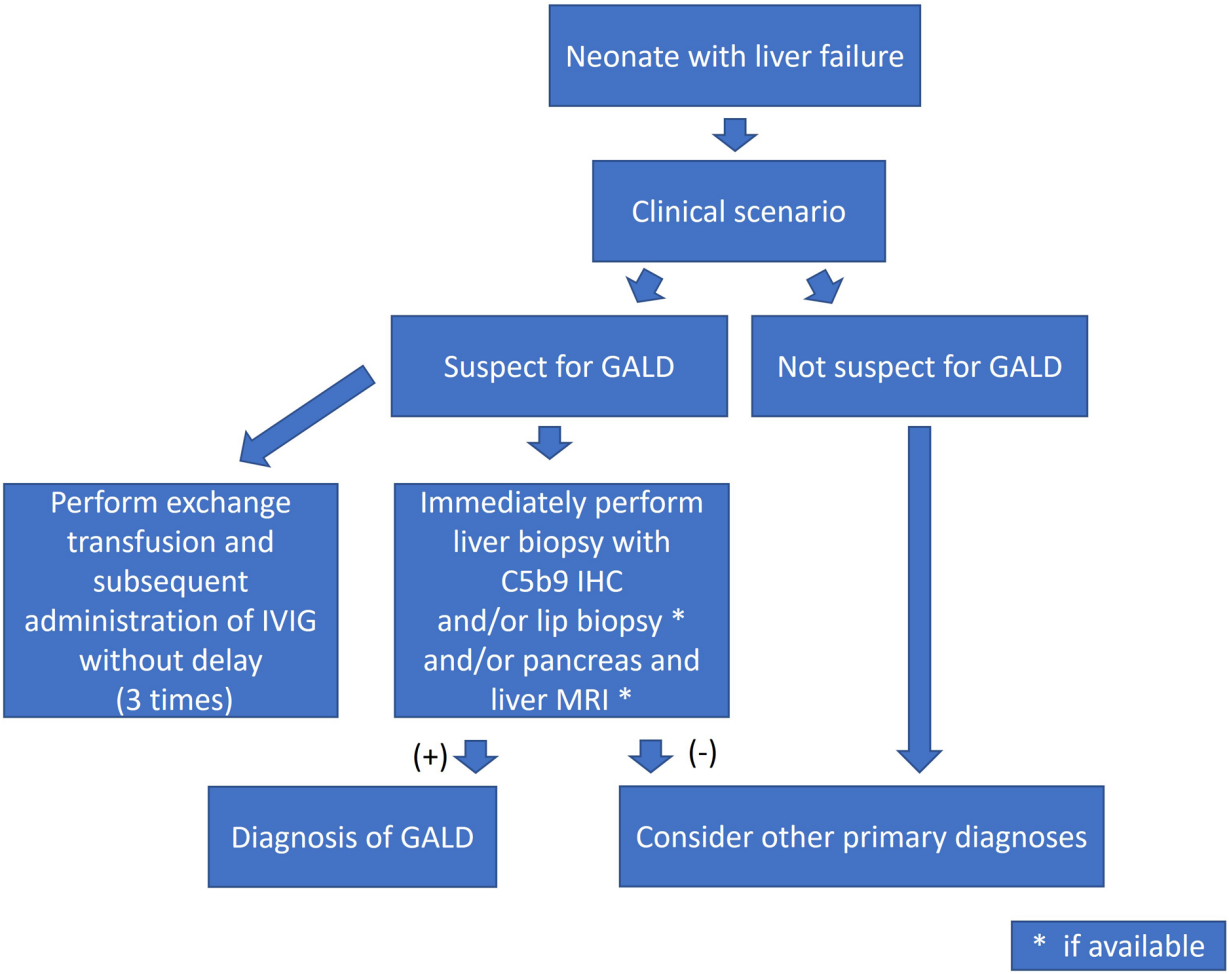
Flow-chart, Erlangen; IVIG, intravenous immunoglobulins, MRI, magnetic resonance imaging, GALD, gestational alloimmune disease, IHC, immunohistochemistry.

In summary, in cases of NALF with clinically and laboratory suspicion of GALD viral hepatitis should be excluded. As especially NALF caused by HSV is associated with high mortality, neonates must be treated immediately at diagnosis of NALF with aciclovir until HSV infection is excluded by PCR. Detection of a persistently patent ductus venosus in a neonate must always be followed by detailed sonographic examination of the liver and clinical-laboratory assessment of liver-injury biomarker values. With regard to GALD, we suggest performing liver and lip mucosa biopsy in one session as soon as possible and expanding diagnostic efforts with MRI of the liver, spleen, and pancreas, if possible and available. Instances of GALD without extrahepatic siderosis thus can be detected and relevant differential diagnosis can be excluded at the same time. This step must be followed by ET and subsequent administration of IVIG without delay. In our experience this procedure should be repeated 3 times (shown in Figure 3). To obtain best clinical outcome patients in whom GALD is suspected, like other infants in NALF, should be immediately transferred to a hepatology center.

## Data Availability

The original contributions presented in the study are included in the article/**Supplementary Material**, further inquiries can be directed to the corresponding author/s.
