# Supplementary material for: Premature birth associated with a favorable course in gestational alloimmune liver disease (GALD): A case report
Source: Front Pediatr. 2023 Mar 16;11:1104530. doi: 10.3389/fped.2023.1104530 (PMC10054034; doi:10.3389/fped.2023.1104530)
Supplement: Supplementary file 1 [file Table1.docx]

Supplementary Material

## Supplementary Figures


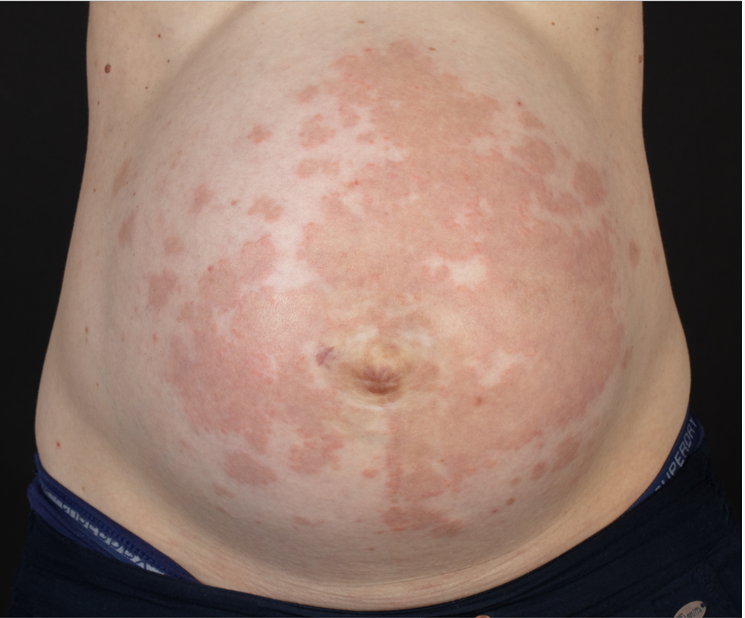


**Supplementary Figure 1.** Pemphigoid gestationis, 20th week of pregnancy.


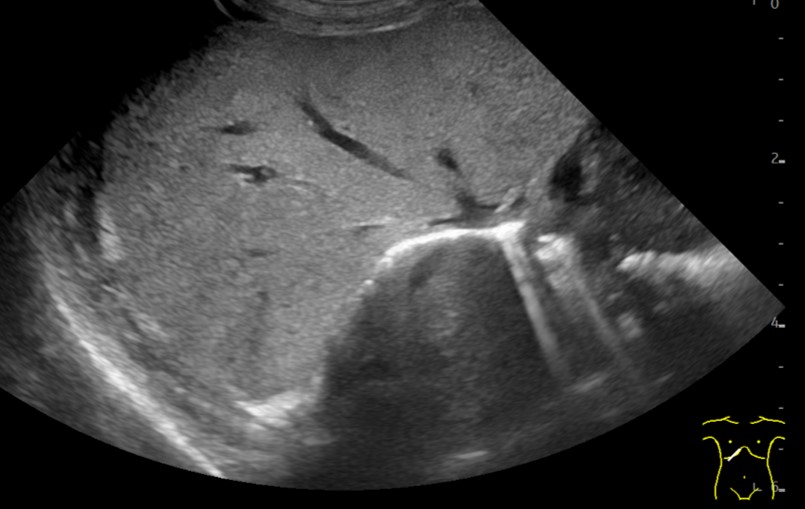
**Supplementary Figure 2.** Sonography - subcostal approach: Liver slightly enlarged, inhomogeneous, hyperechogenic, texture coarsened, contours smooth, aspect consistent with cholestasis and intralobular fibrosis; day 6 of life.


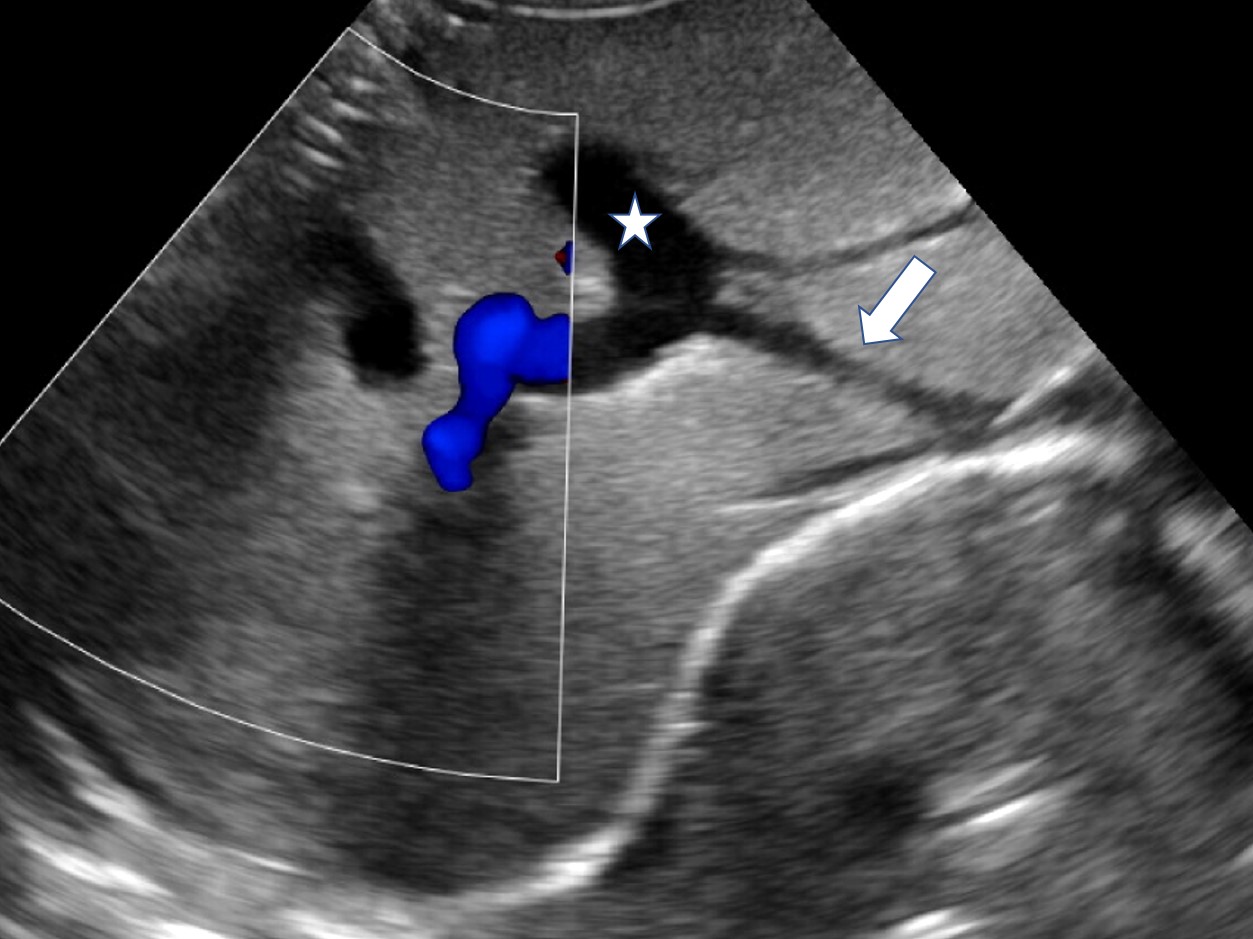


**Supplementary Figure 3.** Sonography - subcostal approach: asterisk: wide open portal vein without signs of periportal fibrosis. Arrow: open ductus venosus arantii: day 5 of life.
